# Conjunctival Melanoma: A Clinical Review and Update

**DOI:** 10.3390/cancers16183121

**Published:** 2024-09-10

**Authors:** Karam Butt, Rumana Hussain, Sarah E. Coupland, Yamini Krishna

**Affiliations:** 1 National Specialist Ophthalmic Pathology Service, Liverpool Clinical Laboratories, Liverpool University Hospitals NHS Foundation Trust, Liverpool L7 8YE, UK; hlkbutt@liverpool.ac.uk (K.B.); s.e.coupland@liverpool.ac.uk (S.E.C.); 2 St Paul’s Eye Unit, Liverpool University Hospitals NHS Foundation Trust, Liverpool L7 8YE, UK; rumana.hussain@liverpoolft.nhs.uk; 3 Department of Eye and Vision Science, Institute of Life Course and Medical Science, University of Liverpool, Liverpool L7 8TX, UK

**Keywords:** conjunctival melanocytic intraepithelial lesions, C-MIL, conjunctival melanoma, ocular oncology, targeted therapy, immunotherapy

## Abstract

**Simple Summary:**

Conjunctival melanoma (Co-M) is a rare and aggressive eye surface cancer. It is often misdiagnosed or overlooked, leading to late diagnosis. Co-M can cause sight loss and even eye loss, impacting quality-of-life. The numbers of new cases globally are rising at alarming rates. There is no standard treatment for Co-M and management varies between eye cancer centres. In ~25% of cases the cancer spreads elsewhere in the body, which can lead to death. The aim of this review is to concisely present what is currently known about Co-M presentation, its development and progression, clinical management and outcomes, and finally summarise future directions for research into novel therapies.

**Abstract:**

Conjunctival melanoma (Co-M) is an aggressive, invasive eye and eyelid cancer. Its global incidence of ~1 in a million is increasing at a rate ratio of ~1.4, but this rises sharply in over 65-year-olds. Although rare, Co-M has a devastating impact on the lives of those who develop it. Co-M is often misdiagnosed or overlooked, leading to vision loss either from the destructive effects of the tumour or side effects of therapy, facial disfigurement from radical surgery, and death from metastases. Due to its rarity, there is limited evidence for diagnosis and management; hence, there is no standardised treatment and not all cases are referred to a specialised ocular oncology centre. Recent progress in cancer immunology and genetics have revolutionised the treatment of cutaneous melanomas, which share some similarities to Co-M. Importantly, a better understanding of Co-M and its precursor lesions is urgently needed to lead to the development of novel targeted and immunotherapies both for local tumour control and disseminated disease. This review aims to provide a comprehensive clinical overview of the current knowledge regarding Co-M, its epidemiology, pathogenesis, presentation, diagnosis and recent changes in the classification of its precursor lesions, management, and recent advances in novel biological therapies for personalised treatment of this disease.

## 1. Introduction

Conjunctival melanoma (Co-M) is a rare, aggressive invasive ocular surface cancer, which is often misdiagnosed or overlooked and causes significant visual disabilities, poor quality of life and even death from metastases. They occur most commonly in fair-skinned populations, with the overall incidence being approximately 0.46 cases per 1,000,000 persons per year [1,2], representing around 0.25% of melanomas at all sites and 5% of all ocular melanomas. It is the second most prevalent malignancy of the conjunctiva after squamous cell carcinoma [3] originating from the basal melanocytes in the conjunctival epithelium. The majority (~70%) of Co-M cases develop from conjunctival melanocytic intraepithelial lesions (C-MIN/PAM with atypia), while others arise from pre-existing naevi or are de novo [3,4].

Previous studies have often grouped Co-M with uveal melanoma as an ocular melanoma: the latter is the most common primary intraocular malignancy in adults, arising in the choroid, ciliary body or iris; however, uveal melanoma is embryologically, biologically and clinically very different to Co-M [5,6,7,8,9]. Unlike uveal melanoma, Co-M is a mucosal melanoma with histological and functional similarities to cutaneous melanoma [10,11] and similar genetic alterations. These include UV-related driver mutations in the *BRAF*, *NF1* and *RAS* genes and copy number variations [6,12,13,14,15,16,17,18,19]. *BRAF* and *NRAS* mutations are present in ~30% and ~14–25% of Co-M, respectively, with the *NRAS* mutant Co-M being associated with higher metastatic risk [13,16]. Transcriptomic studies of Co-M that have focussed on the immune tumour microenvironment have also demonstrated high PDL1-expression and a transcriptomic subtype enriched with immune-system-related genes (immune cell-types) [12,20,21].

Clinically, Co-M most often presents in the bulbar conjunctiva, near the limbus, but can affect any part of the conjunctiva and even invade the neighbouring structures in advanced cases [4,22]. Lesions vary from amelanotic to brown pigmented or even black in colour. There is no standardised treatment; however, management includes surgical excision +/− adjuvant cryotherapy, topical chemotherapy, brachytherapy, proton beam radiotherapy or photon external beam radiation and, in advanced cases with local tissue invasion, radical orbital exenteration [18,22,23,24,25,26,27]. Postoperative complications and tumour recurrence rates are high (33–45%), warranting life-long follow-up [4,28,29,30,31]. Metastases to the lymph nodes are common (~25%) but may also involve the liver, lungs and brain, with ~27% 5-year disease-specific mortality rates [32,33].

Despite recent successes with targeted and immunotherapies in cutaneous melanoma, data on Co-M treated with similar therapies (anti-*BRAF*/anti-*MEK*/anti-*PDL1*) are promising but limited, often stemming from a single patient or small case series with inoperable or advanced disease prior to surgery [34,35,36,37,38].

This review article aims to summarise the current understanding of Co-M and explore new advancements in the knowledge of Co-M pathophysiology, classification, prognostication and its treatment developments.

## 2. Epidemiology

The incidence of Co-M has increased over the last 5 decades [14,39] and ranges from 0.3 to 0.8 per million per year, being highest in Northern Europe and North America. The estimated number of new cases per year is 130 in the USA and 320 in Europe [18]. Populations of Asian or African descent are less commonly affected [2,40,41,42,43]. A study in the US found that fair-skinned people had the highest incidence rate of Co-M, comprising 91.2% of cases compared to 2.4% in patients of Afro-Caribbean descent [44]. However, with majority of data coming from predominantly North America or Europe, there is very limited data on other ethnic groups. Co-M mainly affects patients in their fifth or sixth decades and above; it is rare in children and there is no gender predilection [14,39,40,45,46]. The disease-specific survival rate for Co-M is approximately 82.9% at 5 years and 69.3% at 10 years [30,40].

## 3. Precursor Lesions, Aetiology and Pathogenesis of Co-M

Approximately 70% of Co-Ms arise from conjunctival melanocytic intraepithelial lesions (C-MIL), also known as conjunctival melanocytic intraepithelial neoplasia (C-MIN) or primary acquired melanosis (PAM) with atypia, whilst a smaller proportion develop from pre-existing naevi or are de novo [3,4,47,48]. C-MIL, a precursor or preinvasive disease to Co-M, encompass a spectrum of morphological changes ranging from melanocytic hyperplasia through degrees of melanocytic atypia to melanoma in situ [49]. C-MIL shares the same demographics but may occasionally occur in teenagers and young adults. In a North American analysis of 311 eyes with C-MIL, 96% of the cases were in White individuals and 4% were in Blacks, Hispanics, or Asians, with a patient age range of 15–90 years (mean: 56 years) [23,50].

Both C-MIL and Co-M most frequently develop in the interpalpebral zone, suggesting an association with ultraviolet (UV) radiation [45], with a number of studies revealing UV signatures (C > T transitions) [12,51,52,53]. However, both can also develop in non-sun-exposed sites, but the mechanisms for this are unknown [18].

Driver mutations and copy number variations in multiple chromosomes have been described in Co-M, with high-frequency mutations in the *NF1* (33–50%), *BRAF* (29–46%), *NRAS* (11–26%) and *ATRX* (25%) genes [6,7,12,13,14,15,16,17,54,55,56,57,58,59]. The latter often occurs together with an *NF1* mutation [13]. *NRAS* mutations are associated with higher metastatic risk [13,17]. *TERT* promoter mutations have also been identified in up to 54% of Co-Ms [13,17,60,61] and even in PAM with atypia (~8%) [62]. While activating *TERT* promoter mutations are associated with a poor prognosis, mutually exclusive inactivating *ATRX* mutations appear to be associated with a better prognosis [13,17,36,60]. Cisarova et al. demonstrated that the TGCA-proposed genomic classification of cutaneous melanoma (defined by the most frequently mutated genes: *BRAF, NF1, RAS* and triple wild-type) was also applicable to Co-M [12]. A summary of the common gene mutations is presented in Table 1. Other rarer mutations have been reported in the genes, such as *CTNNB1, ACSS3, RET, TP53, CKIT, TET2, CDKN2A, MAPK2, RAC1, MET, SF3B1, GNAQ and GNA11* [12,13,16,63,64,65]. Transcriptomic studies in Co-M focusing on the immune tumour microenvironment have demonstrated high PDL1-expression, and a transcriptomic subtype enriched with immune-system-related genes (immune cell-types) [12,20,21]

## 4. Clinical Presentation and Assessment

**Figure 1:**
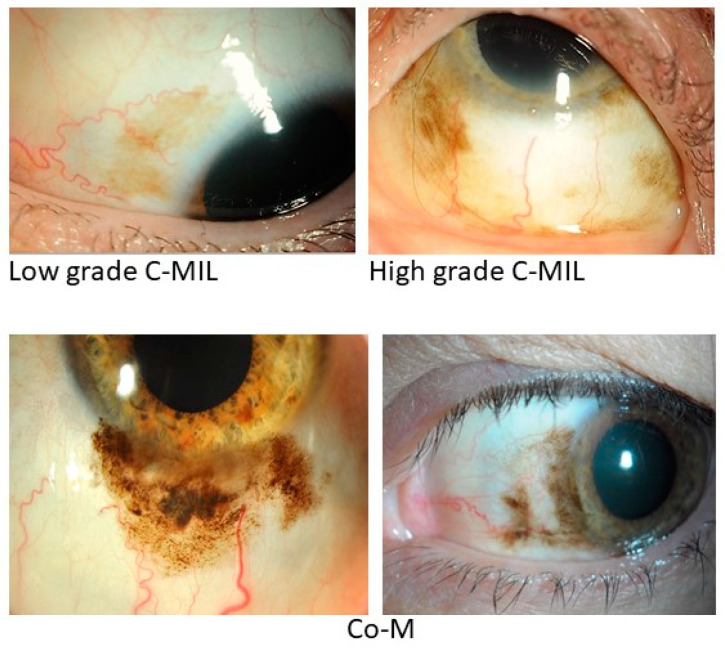
Anterior segment photographs of low- and high-grade C-MIL (preinvasive disease) and Co-M (invasive disease). The actual grading was confirmed on histomorphological assessment.

Co-M is often unilateral and can affect any part of the conjunctiva, but commonly presents on the bulbar surface, near the limbus (Figure 1). Invasion of the cornea, eyelid, sclera or orbit may occur in advanced tumours [4]. The lesions vary in size, shape and colour. Nodular masses may be well circumscribed, while flat lesions may have irregular, ill-defined margins, especially where there is adjacent C-MIL. Their colour may range from amelanotic (pinkish) to various shades of brown or even black and be patchy or mixed within the one lesion [70]. Patients may present on noticing a mass with/without pigmentation on their eye but can also have significant visual morbidities, such as irritation/burning with redness and reflex tearing, dry eye, pain, vision disturbance, double vision or vision loss [4,71,72].

The differential diagnoses of Co-M include benign conjunctival nevi, extraocular extension of uveal melanoma or melanocytoma (black lesions), pigmented conjunctival squamous cell carcinoma or, very rarely, metastasis of cutaneous melanoma. In amelanotic lesions, the differentials also include conjunctival squamous intraepithelial neoplasia, squamous cell carcinoma or lymphoma [72]. Lack of cysts (often observed in conjunctival nevi), tumour haemorrhage, large/deep tumours, tortuous feeder vessels, adherence to underlying and invasion into surrounding structures, and multifocal lesions favour melanoma over conjunctival nevus [73]. Histological assessment in a specialist centre regularly reporting ophthalmic specimens is essential for accurate diagnosis and grading.

C-MIL are unilateral but most often multifocal, can involve any part of the conjunctiva (bulbar, limbal, forniceal and palpebral, in order of decreasing frequency) (Figure 1) and may extend to the caruncle, plica and cornea. They appear as mobile, flat, irregular brown-pigmented conjunctival discolorations that may change over time [14,22,23,39,40,50]. Rarely, C-MIL can be amelanotic, making it diagnostically challenging [22,23]. Differential diagnoses of C-MIL include benign epithelial melanosis, oculodermal melanocytosis, conjunctival nevi, Co-M, pigmented conjunctival squamous intraepithelial neoplasia [74], Addison’s disease [75], post-inflammatory hyperpigmentation and conjunctival tattooing [76].

Imaging of all conjunctival melanocytic lesions involves regular anterior segment photo-documentation (including with eversion of eyelids), slit lamp biomicroscopy and possibly ultrasound (to estimate tumour thickness or look for orbital involvement). There are recent developments in anterior segment optical coherence tomography [77,78] and in vivo reflectance confocal microscopy [79]. Where Co-M has locally extended into the eyelid, and particularly if there is any suspicion of orbital or nasolacrimal invasion, MRI has a critical role in the assessment; diffusion and perfusion-weighted imaging can help in differentiating Co-M from other eyelid masses [80].

## 5. Histomorphological Features

**Figure 2:**
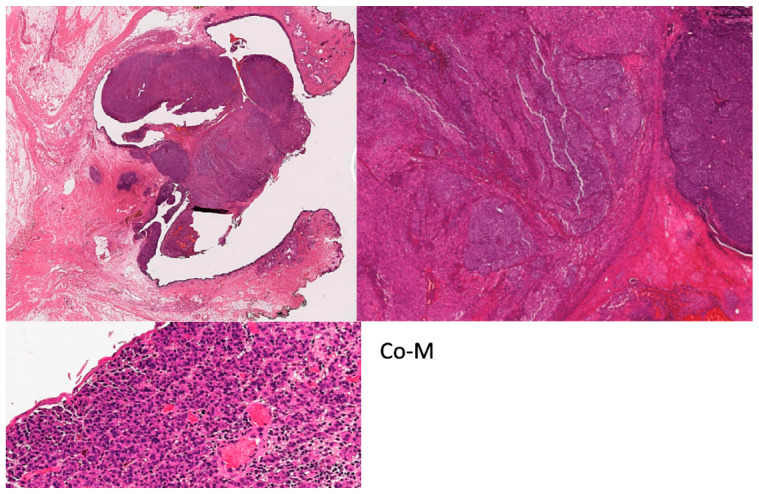
Co-M histological micrographs. Haematoxylin and eosin staining photomicrographs showing an orbital exenteration specimen with Co-M at low magnification (**top left**), medium (**top right**) and higher magnification (**bottom left**).

Co-M shares similar histomorphological features to those of cutaneous or other mucosal invasive melanomas [81]. The intraepithelial (radial) component can be nested, pagetoid, lentiginous or, rarely, absent. It can also extend beyond the invasive component and involve adjacent structures. The invasive stromal (vertical) component can comprise nests or sheets of atypical melanocytes with cytomorphology ranging from small naevoid-type cells to highly atypical pleomorphic melanocytes with spindle or epithelioid types. Nuclei can be hyperchromatic with inconspicuous nucleoli or vesicular with prominent eosinophilic nucleoli (Figure 2). Ulceration, mitotic activity, angiotropism, satellites in transit metastases and neurotropism may be seen. A pre-existing nevus also may be present. Melanophages and variable lymphocytic infiltrate are also often observed. Increased mitoses (>5.5 mitoses/mm^2^) have been associated with nodal metastasis; ulceration and a greater tumour thickness have been associated with increased mortality, similar to skin melanoma [82,83].

The key histomorphological features in C-MIL are the increased cellularity/hyperplasia of the intraepithelial conjunctival melanocytes with increasing cytological atypia, but the basement membrane remains intact [81,84,85]. The spectrum of cytological features ranges from small melanocytes with nuclear hyperchromasia and scant cytoplasm to severely atypical large pleomorphic epithelioid cells with ample cytoplasm and prominent eosinophilic nucleoli. The range of atypical architectural patterns include linear hyperplasia of the basal melanocytes to a confluent lentiginous spread, intraepithelial nests, pagetoid growth and full-thickness epithelial involvement by atypical melanocytes, i.e., melanoma in situ. Nests, pagetoid spread and confluent growth extend upward from the basal epithelium, displacing squamous and/or goblet cells; however, there should be no evidence of invasive growth [49,81,84,85]. Epithelioid cell morphology with cytological atypia, nesting and pagetoid spread are associated with an increased risk of recurrence and progression to Co-M [23,81,84,86,87].

Terminologies more commonly used to classify these lesions include PAM with atypia and C-MIN and the most recently validated C-MIL [85,88]. Various grading or scoring systems have been used for C-MIL [22,75,84,85,87,89,90,91]. The grading of the cytological atypia in PAM (mild, moderate or severe), which was similar to those used in the skin and other mucosal sites [84,86], suffered from poor reproducibility between pathologists. The C-MIN scoring system was based on architectural features (i.e., horizontal and vertical spread) and cytological atypia but could be time consuming and complex [89].

**Figure 3:**
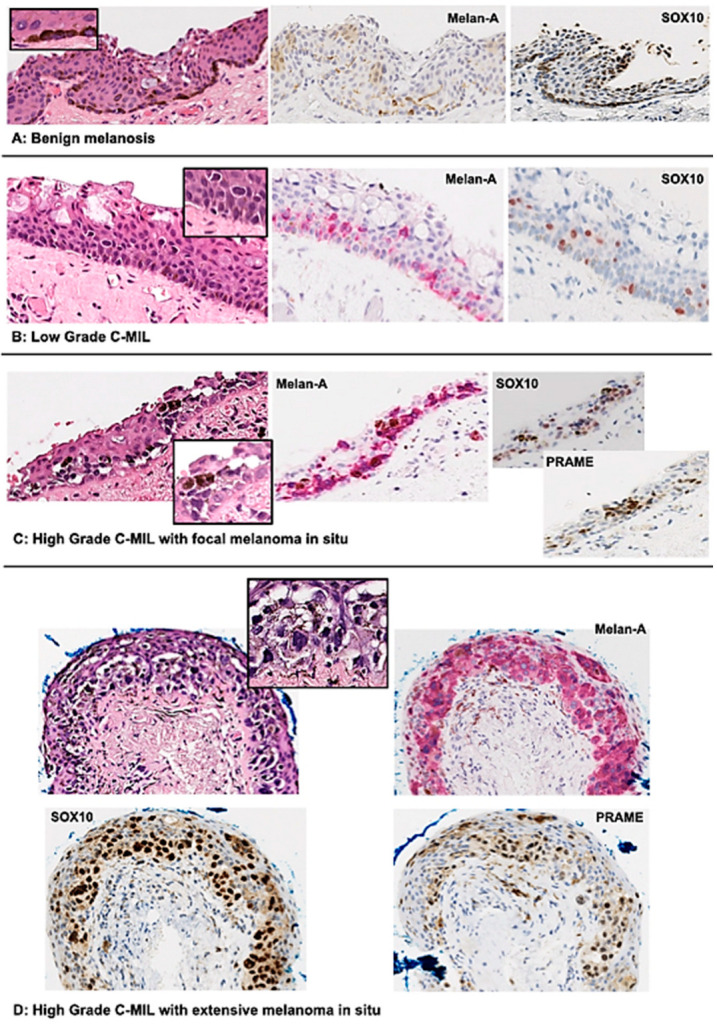
Photomicrographs representing the C-MIL grading system. Haematoxylin and eosin section with corresponding immunohistochemistry for each of the C-MIL scoring grades [88].

In 2018, the fourth ‘WHO Classification of Eye Tumours’ proposed the C-MIL classification, simplifying the grading of these lesions and capturing their risk of disease progression to invasive melanoma [92]. This comprised: (1) low-grade C-MIL (corresponding to PAM with or without mild atypia or C-MIN scores 1–2; (2) high-grade C-MIL (PAM with moderate to severe atypia or C-MIN 3–5); and (3) conjunctival melanoma in situ (PAM with severe atypia involving > 75% of the epithelium or a C-MIN score > 5). The system was validated in 2021 and it was found that all three classification systems (C-MIL, C-MIN and PAM) had comparable accuracy in their ability to identify lesions with potential for recurrence [85]. In 2022, the editorial panel of the fifth edition decided to revise the classification scheme because the low-grade C-MIL in the fourth edition incorporated both non-neoplastic and neoplastic melanocytic proliferations. This led to the current system as summarised in Table 2 [49]. This was validated by a large international collaborative study and found to have substantial interobserver agreement, good reproducibility, be predictive of recurrence and invasive disease and, importantly, inform clinical treatment thresholds [88]. Photomicrographs demonstrating the C-MIL scoring grades are presented in Figure 3 [88].

All conjunctival melanocytic lesions immunohistochemically show *MelanA (MART1), S100, SOX10, HMB45* (the latter should only be present in superficial areas of naevi) and *MITF* (Melanocyte Inducing Transcription Factor). Nuclear expression of *PRAME* (Preferentially expressed Antigen in Melanoma), cyclin D1 positivity and loss of p16 on immunohistochemistry can also be helpful in distinguishing Co-M from naevi or low-grade C-MIL (both *PRAME* and cyclin D1 negative, p16 positive) [57,88,93,94,95,96]. PD-L1 may be expressed [97].

Melanoma in situ and Co-M are staged by the AJCC/UICC TNM eighth edition system, which has been validated for the development of metastasis and survival [98,99,100].

## 6. Treatment and Prognosis

Approximately 70% of all Co-M arises from high-grade C-MIL [75]; hence, there is an absolute clinical need to know when to treat patients [22,23,88]. There is no standard-of-care treatment for C-MIL or Co-M; consequently, management varies considerably between ophthalmic and specialised ocular oncology centres. This includes: surgical excision (wide local) +/− amniotic membrane allograft and +/− adjuvant cryotherapy, topical chemotherapy (mitomycin C, 5-fluorouracil or interferon alpha-2b), radiotherapy (brachytherapy, proton beam or photon external beam) or radical orbital exenteration for advanced cases with local tissue invasion [4,22,23,32,72].

The reported usefulness of sentinel lymph node biopsy (SLNB) is variable (in terms of clinical management and sensitivity of pickup) but has been shown to be effective for Co-Ms > 2 mm thickness and/or >10 mm in diameter [101].

The postoperative complication rate (vision loss, scarring, limbal stem cell failure, ulceration/non-healing defects, etc.) and risk of tumour recurrence are very high (33–61%), warranting close life-long follow-up [4,28,29,30,31,72]. Lymph node metastases are common (~25–52%; preauricular, parotid, submandibular and/or cervical nodes, depending on Co-M location) but metastasis may also involve the liver, lungs and brain (11–42%). Indicators/risk factors of poor prognosis for nodal and systemic metastases include a non-epibulbar locations, ulceration and increased tumour thickness [32,33,47,48,82,83]. The 5-year and 10-year disease-specific mortality rates are ~14–27% and 25–35%, respectively [4,30,32,33,47,48,72,102]. Similarly to the risk of metastases, tumour-related death has been associated with a de novo origin, non-bulbar conjunctival location, nodular growth, multifocal lesions and sentinel lymph node positivity [4,30,43,83,103].

The use of genetics for prognostication in Co-M is currently limited. However, as mentioned above, Co-M has genetic alterations similar to those of cutaneous melanoma, and advances in characterising Co-M genetics are offering insight into potential targeted therapies that are already in use for the treatment of cutaneous melanoma. Data on Co-M (anti-*BRAF*; anti-*MEK*; anti-*PD-L1*) with targeted/immunotherapies have shown promising results but are limited, with only those from small case series or single case studies in patients with inoperable disease or as first-line therapy prior surgery in advanced cases [18,34,35,38,104]. A summary of the targeted, immune checkpoint inhibitor and combination therapies is presented in Table 3 [34,35,38,51,105,106,107,108,109,110,111,112,113,114,115,116,117]. A Phase 2 clinical trial using a combination of axitinib and nivolumab in untreated advanced or metastatic mucosal melanoma (head and neck and conjunctival; NCT05384496) is underway.

## 7. Future Direction and Conclusions

Co-M is a rare, aggressive, invasive eye and eyelid cancer with increasing global incidence. Given the rarity of Co-M, international collaboration is pivotal to obtain sufficient numbers in order to progress translational research and enlist Co-M patients into clinical trials. The recent developments in cancer genetics and immunology present exciting new frontiers for better understanding Co-M pathogenesis and, importantly, provide new targets for therapy. Insight into the molecular drivers for Co-M development and its integration with clinical and histomorphological evaluation will allow earlier diagnosis, improve risk stratification and prognostication, and identify patients for specific therapies (i.e., ‘personalised/precision medicine’). This will further enable the development of clear management guidelines and enrolment into targeted therapies earlier than current practice, facilitating improved outcomes in this rare disease.

## Figures and Tables

**Table 1:** Common genetic mutations in the conjunctival naevi, primary acquired melanosis (PAM) without atypia, PAM with atypia, and conjunctival melanoma (Co-M). The number of cases and their respective percentages are presented. NAD = No available data.

	Conjunctival Naevi (%)	PAM without Atypia (%)	PAM with Atypia (%)	Prevalence in Co-M (%)
*BRAF*	14/28 (50%) [54] 13–23 (56%) [58] 7/37 (19%) [55] 9/12 (75%) [14] 15/35 (43%) [57]	0/11 (0%) [54] 0/17 (0%) [55]	0/4 (0%) [54] 0/13 (0%) [55] 2/8 (25%) [14]	4/15 (27%) [64] 3/21 (14%) [5] 12/22 (55%) [7] 23/78 (29%) [56] 2/5 (40%) [54] 10/39 (26%) [55] 39/111 (35%) [14] 4/53 (8%) [65] 31/101 (31%) [13] 16/47 (34%) [16] 13/28 (46%) [17] 4/14 (29%) [12] 23/78 (29%) [60] 11/31 (35%) [57] 5/22 (23%) [59] 16/63 (25%) [15] 7/15 (47%) [66] 3/12 (25%) [67] 10/38 (26%) [61] 1/8 (13%) [51]
*NRAS*	9/23 (39%) [58]	NAD	NAD	0/11 (0%) [64] 14/78 (18%) [56] 25/95 (26%) [13] 5/47 (11%) [16] 6/28 (21%) [17] 1/14 (7%) [12] 11/63 (17%) [15] 4/15 (27%) [66] 3/12 (25%) [67] 5/38 (13%) [61] 3/8 (38%) [51]
*KIT*	0/5 (0%) [63]	NAD	1/3 (33%) [63]	1/13 (8%) [64] 0/42 (0%) [56] 0/8 (0%) [63] 6/53 (11%) [65] 2/47 (4%) [16] 2/28 (7%) [17]
*TERT*	0/56 (0%) [62]	0/14 (0%) [62]	2/25 (8%) [62]	12/38 (32%) [61] 16/39 (41%) [62] 20/47 (43%) [60] 15/24 (54%) [17] 9/14 (64%) [12] 34/78 (43%) [60] 7/15 (47%) [66] 26/58 (45%) [68]
*NF1*	NAD	NAD	NAD	21/63 (33%) [15] 29/74 (39%) [13] 7/14 (50%) [12] 17/47 (36%) [16] 3/15 (20%) [66] 1/5 (20%) [69] 3/8 (38%) [51]
*ATRX*	0/16 (0%) [68] *	0/6 (0%) [68] *	2/5 (40%) [68] *	17/68 (25%) [13] 5/8 (63%) [51] 8/59 (14%) [68] * 1/5 (20%) [69]

* ATRX protein detection by immunohistochemistry.

**Table 2:** The 2022 WHO classification of conjunctival melanocytic intraepithelial lesions (C-MIL) [49].

WHO	Acceptable Alternative Terminology	Increased Cellularity	Histologic Features	Progression Risk to Invasive Melanoma
Not applicable	Benign melanosis C-MIN (grades 0–1) PAM without atypia	No/minimal	Conjunctival hypermelanosis (increased pigment in epithelial cells without melanocytic hyperplasia or atypia). Slight or focal melanocytic hyperplasia without atypia (parabasal melanocytes with condensed round nuclei, smaller than basal epithelial cell, inconspicuous nucleoli and inconspicuous cytoplasm) may be seen.	None
Low-grade C-MIL	PAM with mild atypia C-MIN (grades 2–4)	Yes	Predominantly basilar melanocytic proliferation with low-grade atypia (dendritic or small to moderate size polyhedral, usually non-epithelioid melanocytes with round to irregular nuclear contours, often nuclear hyperchromasia, inconspicuous nucleoli and inconspicuous or scant cytoplasm).	Lower
High-grade C-MIL	PAM with moderate to severe atypia C-MIN (grade 5–10)	Yes	More confluent basilar and significant non-basilar proliferation of melanocytes with high-grade atypia (moderate to severe), evidence of intraepithelial nested and/or pagetoid growth and epithelioid cell cytomorphology.	Higher
High-grade C-MIL	Melanoma in situ	Yes	The term melanoma in situ may be used for (1) the most atypical high-grade C-MILs involving close to full thickness of the epithelium or (2) histologically obvious melanomas without documented evidence of subepithelial invasion.	Highest

**Table 3:** Reported cases of targeted, immune checkpoint inhibitor and combination therapies used in primary and metastatic Co-M.

Study	Patient	Co-M	Primary Treatment	Agent(s) Used	Dosage (s)	Outcome	Adverse Reactions
Indicated for primary Co-M							
[105]	80y, female	BRAF mutation	Exenteration (rejected)	Vemurafenib	-	Successful tumour response Tumour decreased in size	8 kg weight loss Nausea, vomiting, headache
[35]	94y, Female	Bulbar to eyelid	None (rejected exenteration)	First—Pembrolizumab Second—Pembrolizumab and ipilimumab	Pembrolizumab, 200 mg; Ipilimumab, 1 mg/kg	Progression Died after 5 months	None reported
[35]	76y, Male	Recurrence Cornea to eyelid	Local treatments and topical interferon-alpha chemotherapy	First—ipilimumab Second—Pembrolizumab Third—Pembrolizumab and IFN-alpha	Pembrolizumab, 2 mg/kg every 3 weeks	Ipilimumab—no response Pembrolizumab—minimal response, then complete with IFN-alpha	Ipilimumab—adrenal insufficiency Pembrolizumab—dermatitis
[35]	84y, Female	Recurrence Cornea to eyelid	Excision Cryotherapy Topical mitomycin Eye plaque brachytherapy	First—Pembrolizumab Second—Pembrolizumab and ipilimumab Third—Pembrolizumab, ipilimumab and IFN-alpha	Pembrolizumab, 200 mg; Ipilimumab, 1 mg/kg; IFN-alpha, 3 million units per eyelid	Pembrolizumab—minimal success Pembrolizumab and ipilimumab—progression	None reported
[106]	53y, Female	Bulbar to tarsal	None	Pembrolizumab	200 mg every 3 weeks	Complete reduction of pigment and disease free 12 months of follow up	Cutaneous pruritus
[51]	70s y, Female	BRAF v600e Diffuse bulbar and anterior orbit, lower eyelid margin Left eye	None	Dabrafenib and trametinib	-	Regression after 3 months then excision No local recurrence Developed metastasis 1 year later	None reported
[107]	61y, female	Right upper eyelid and superior bulbar conjunctiva BRAF v600e mutation Recurrence	Excision Cryotherapy	Dabrafenib and trametinib Changed to vemurafenib after 1.5 months Changed to pembrozilumab after 3.5 months Vemurafenib restarted after 2 months in addition to cobimetinib	-	Nearly complete resolution 1-month post-treatment No evidence of distant spread of disease 23 months later	Dabrafenib and trametinib—nausea and vomiting
[108]	52y, male	Right eye BRAF v600e Mutlifocal recurrence	Incisional biopsy	Dabrafenib Trametinib	Dabrafenib twice daily 150 mg Trametinib daily 2 mg	Complete resolution after 10 months Metastasis free after 15 months	Fevers Elevated liver enzymes
[109]	60s, male	Multifocal recurrence, orbital and intraocular invasion	Excision Cryotherapy	Pembrolizumab	150 mg every 3 weeks—18 cycles	Alive without disease	None reported
Indicated for metastatic disease							
[110]	45y, male	Metastatic Co-M (nodal, subcutaneous, pulmonary, osseous) BRAF mutation v600e	Resection	Vemurafenib	960 mg twice daily	Improvement in pain and subjective tumour regression after 1 month	Disease progression 2 months into treatment. Enlarged paraspinal mass.
[111]	53y, female	Metastatic Co-M (orbit, parotid gland, lung, brain) BRAF mutation v600e	Excision Cryotherapy Mitomycin eye drops Enucleation	Vemurafenib	960 mg twice daily, then changed to 720 mg twice daily due to skin rash	Initially good response and reduction of mets; after 4 months reappearance of mets and death	Skin rash
[38]	70y, male	Metastatic Co-M (parotid gland and lymph node) BRAF mutation v600e	Excisional biopsy	Dabrafenib Trametinib	Dabrafenib (150 mg twice daily) Trametinib (2 mg daily)	Reduction of lymph node metastasis activity	Fever
[112]	59y, female	Metastatic Co-M (Oropharyngeal wall) BRAF mutation v600	Excision	Vemurafenib	960 mg twice daily, then later changed to 480 mg twice daily due to diarrhoea and skin rash	Full symptomatic recovery after 1 month Developed breast cancer	Arthralgia, diarrhoea, skin rash
[112]	51y, Male	Co-M recurrence with metastasis (lymph) No BRAF mutation	Excision Lymphadenectomy	Pembrolizumab	2 mg/kg every 3 weeks	Complete resolution of subcutaneous lesions	None noted, patient on complete remission
[34]	68y, Female	Co-M recurrence Metastasis—lung BRAF v600e mutation	Resection Topical mitomycin C Exenteration, sentinel lymph node biopsy	First—Pembrolizumab Second—Ipilimumab and dacarbazine	Pembrolizumab, 2 mg/kg every 3 weeks; Ipilimumab, 3 mg/kg; Dacarbazine, 800–1000 mg/m^2^	Pembrolizumab—stable at 6 months	Ipilimumab and dacarbazine—hepatotoxicity
[34]	58y, Female	Co-M recurrence to orbit Metastasis—lung and liver	Multiple resections Orbital exenteration	nivolumab	3 mg/kg every 2 weeks	Complete resolution or orbit and metastasis lesions	Elevated liver enzymes
[34]	28y, Female	Co-M recurrence Metastasis—breast, lung and bone	Excision Cryotherapy Topical mitomycin C	nivolumab	3 mg/kg every 2 weeks	Complete resolution	None reported
[34]	47y, Female	Co-M recurrence Metastasis—lung	Excision Cryotherapy Radiotherapy Topical interferon Mitomycin C	nivolumab	3 mg/kg every 2 weeks	Resolution of lung metastasis and free from disease 7 months after nivolumab	Diarrhoea
[34]	74y, Male	Co-M recurrence Metastasis—lung	Multiple excision	nivolumab	3 mg/kg every 2 weeks	Decrease in tumour size Disease free 1 month after nivolumab	Colitis
[113]	72y, Male	Recurrent Co-M Metastasis—Lung and lymph	Debulking and sentinel lymph node biopsy Radioactive iodine 125	Ipilimumab	3 mg/kg every 3 weeks	Satisfactory response to treatment and excellent local tumour control	Mild fatigue
[35]	72y, Female	Epibulbar BRAF v600k Metastasis—liver, lung, bone, skin, lymph node	Local excision and topical chemotherapy	Ipilimumab and nivolumab	Ipilimumab, 3 mg/kg; Nivolumab, 1 mg/kg	Resolution of subcutaneous nodulesReduction of systemic tumour burden	Hepatotoxicity Colitis
[35]	76y, Female	NRAS mutation Metastasis—lymph, skin	Excision Cryotherapy Topical mitomycin chemotherapy	First—ipilimumab Second—ipilimumab Third—Pembrolizumab	Ipilimumab, 3 mg/kg; Pembrolizumab, 200 mg	Ipilimumab—new skin metastases and lymph metastases	None reported
[114]	71y, Male	Co-M recurrence BRAF v600e Metastasis—bone and liver	Excision Cryotherapy Vemurafenib	Nivolumab Dabrafenib and trametinib	-	Died 24 months after combined therapy	Vemurafenib—keratinous nodules
[114]	72y, Male	Bulbar BRAF v600e Co-M with lymph node metastasis	Excision Cryotherapy Mitomycin C eye drops	Dabrafenib and trametinib	-	No signs of recurrence after 6 months	None reported
[106]	66y, Male	Fornix and orbit Metastasis—lung and liver	None	Ipilimumab and nivolumab	-	Resolution of lesion and good response to mets	Pituitary failure
[115]	60y, female	Recurrent conjunctival melanoma NRAS mutation Liver metastasis	Excision at 3 and 7 months Cryotherapy	Ipilimimab and nivolumab—2 cycles Nivolumab—3 cycles Pembrolizumab—9 cycles	Ipilimimab 3 mg/kg Nivolumab, 1 mg/kg Nivolumab, 240 mg every 2 weeks 2 cycles, then 480 mg every 4 weeks Pembrol zumab, 200 mg	Remained stable after 2 years	Ipilimimab and nivolumab—hepatitis Nivolumab—infusion reaction
[116]	89y, female	Recurrence BRAF v600e right conjunctiva Distant metastasis	Resection	Encorafenib Binimetinib	Encorafenib 450 mg once daily; Binimetinib 45 mg twice daily	Reduction in size of primary tumour and distant metastases after 6 months	None reported
[117]	60y, female	Recurrence and metastasis to lymph and nasal cavity (BRAF negative)	Excisional biopsy Cryotherapy	Ipilimumab Nivolumab	Ipilimumab 1 mg/kg; Nivolumab 3 mg/kg	25% reduction in size of nasal cavity mass, which persisted after 16 months No evidence of recurrence at one-year follow-up	None reported
