# Correction: Butt et al. Conjunctival Melanoma: A Clinical Review and Update. *Cancers* 2024, *16,* 3121

**DOI:** 10.3390/cancers16223721

**Published:** 2024-11-05

**Authors:** Karam Butt, Rumana Hussain, Sarah E. Coupland, Yamini Krishna

**Affiliations:** 1 National Specialist Ophthalmic Pathology Service, Liverpool Clinical Laboratories, Liverpool University Hospitals NHS Foundation Trust, Liverpool L7 8YE, UK; hlkbutt@liverpool.ac.uk (K.B.); s.e.coupland@liverpool.ac.uk (S.E.C.); 2 St Paul’s Eye Unit, Liverpool University Hospitals NHS Foundation Trust, Liverpool L7 8YE, UK; rumana.hussain@liverpoolft.nhs.uk; 3 Department of Eye and Vision Science, Institute of Life Course and Medical Science, University of Liverpool, Liverpool L7 8TX, UK

## Missing Acknowledgement

The authors would like to include the following acknowledgment in the publication [1]:

All authors are very grateful and extend their thanks to Heinrich Heimann, who is a Co-Lead at the Liverpool Ocular Oncology Centre, where these rare ocular tumours are managed. Heimann has been caring for these patients for the last 15 years and is actively involved in many clinical trials for various ocular tumours.

The authors state that the scientific conclusions are unaffected. This correction was approved by the Academic Editor. The original publication has also been updated.
